# Immunophenotyping of Waldenströms Macroglobulinemia Cell Lines Reveals Distinct Patterns of Surface Antigen Expression: Potential Biological and Therapeutic Implications

**DOI:** 10.1371/journal.pone.0122338

**Published:** 2015-04-08

**Authors:** Aneel Paulus, Kasyapa S. Chitta, Paul K. Wallace, Pooja P. Advani, Sharoon Akhtar, Maja Kuranz-Blake, Sikander Ailawadhi, Asher A. Chanan-Khan

**Affiliations:** 1 Department of Cancer Biology, Mayo Clinic, Jacksonville, Florida, United States of America; 2 Department of Flow and Image Cytometry, Roswell Park Cancer Institute, Buffalo, New York, United States of America; 3 Division of Hematology and Oncology, Department of Internal Medicine, Mayo Clinic, Jacksonville, Florida, United States of America; Mie University Graduate School of Medicine, JAPAN

## Abstract

Waldenströms macroglobulinemia (WM) is a subtype of Non-Hodgkin’s lymphoma in which the tumor cell population is markedly heterogeneous, consisting of immunoglobulin-M secreting B-lymphocytes, plasmacytoid lymphocytes and plasma cells. Due to rarity of disease and scarcity of reliable preclinical models, many facets of WM molecular and phenotypic architecture remain incompletely understood. Currently, there are 3 human WM cell lines that are routinely used in experimental studies, namely, BCWM.1, MWCL-1 and RPCI-WM1. During establishment of RPCI-WM1, we observed loss of the CD19 and CD20 antigens, which are typically present on WM cells. Intrigued by this observation and in an effort to better define the immunophenotypic makeup of this cell line, we conducted a more comprehensive analysis for the presence or absence of other cell surface antigens that are present on the RPCI-WM1 model, as well as those on the two other WM cell lines, BCWM.1 and MWCL-1. We examined expression of 65 extracellular and 4 intracellular antigens, comprising B-cell, plasma cell, T-cell, NK-cell, myeloid and hematopoietic stem cell surface markers by flow cytometry analysis. RPCI-WM1 cells demonstrated decreased expression of CD19, CD20, and CD23 with enhanced expression of CD28, CD38 and CD184, antigens that were differentially expressed on BCWM.1 and MWCL-1 cells. Due to increased expression of CD184/CXCR4 and CD38, RPCI-WM1 represents a valuable model in which to study the effects anti-CXCR4 or anti-CD38 targeted therapies that are actively being developed for treatment of hematologic cancers. Overall, differences in surface antigen expression across the 3 cell lines may reflect the tumor clone population predominant in the index patients, from whom the cell lines were developed. Our analysis defines the utility of the most commonly employed WM cell lines as based on their immunophenotype profiles, highlighting unique differences that can be further studied for therapeutic exploit.

## Introduction

Waldenströms Macroglobulinemia (WM) is a lymphoplasmacytic lymphoma that is characterized by small malignant lymphocytes, plasmacytoid lymphocytes and/or plasma cells that predominantly invade the bone marrow and secrete immunoglobulin-M (IgM).[1] As a result of tumor cell infiltration, patients with WM can present with clinical features of lymphadenopathy, hepatosplenomegaly or pancytopenia. Moreover, WM cells are known to secrete large amounts of IgM resulting in hyperviscosity and end organ damage.[2, 3] WM is a relatively rare malignancy, with an estimated 1500 new cases diagnosed per year in the United States and an incidence of 3 to 5 persons per million persons per year.[4, 5] Due to its rarity, immunophenotypic ambiguities related to the WM tumor compartment being comprised of different populations of B-cells, and scarcity of reliable preclinical models, WM remains a challenging and incurable hematologic malignancy.[6] Although limited in number, WM cell line models have indeed allowed for rigorous examination of disease mechanisms along with providing a platform for testing anti-WM therapeutics.

The optimal use of a preclinical model system can be derived upon its comprehensive characterization. Molecular assessment through whole exome sequencing, global transcriptome profiling as well as micro-RNA (miRNA) and methylation profiling is now routinely performed on cell lines with the results cataloged in online databases.[7] However, efforts to define and catalog the immunophenotypic features of preclinical models (and particularly WM) have been lacking. The total phenotypic makeup (molecular and immunophenotypic) carries far greater potential for precisely defining a models functional utility, particularly when testing targeted therapies such as monoclonal antibodies, which rely on engagement with external cell surface receptor/antigens to exert their effects internally.

The presence or absence of cell surface antigens typically remains consistent, in contrast to gene or miRNA expression, which are highly contextual and change in response to a variety of stimuli, including therapy induced stress. However, it has been reported that the WM surface marker profile can shift over time from that of a predominantly monotypic B-lymphocytic type towards one more reminiscent of a plasma cell population, in response to treatment with various chemoimmunotherapeutics.[8] This shift in cell populace is reflected by loss of characteristic B-lymphocyte surface antigens (CD19, CD20) and acquisition/overexpression of plasma cell markers (CD38, CD138), which can be detected by flow cytometry or immunohistochemistry.[8, 9]

We have previously reported on development and establishment of the RPCI-WM1 human WM cell line, which is CD19- and CD20- and was developed from a WM patient with terminal disease.[10] RPCI-WM1 constitutes a unique model amongst the other WM cell lines (BCWM.1 and MWCL-1) due to loss of CD19/20[10, 11] and as such may derive its origins from a tumor population that was predominately comprised of plasma or plasmacytoid cells. Extending on this original observation, we conducted a comprehensive immunophenotyping analysis to profile the presence or absence of WM and hematopoietic lineage (non-WM) CD antigens in RPCI-WM1 as well as the BCWM.1 and MWCL-1 models in a comparative manner.

## Materials & Methods

### Cell lines, cell culture and reagents

Human Waldenström macroglobulinemia cell lines were used in this analysis and maintained as previously described.[12] The BCWM.1 and MWCL-1 cell lines were kindly gifted to us from Dr. Steven Treon (Dana Farber Cancer Institute, Harvard, MA) and Dr. Steven Ansell (Mayo Clinic, Rochester, MN).[13, 14] The RPCI-WM1 model was established and developed as previously reported.[10] All cell lines were cultured in RPMI-1640 containing 10% FBS and penicillin (100U/ml) and streptomycin (100ug/ml). Culture medium was replaced every three days. Cell viability was maintained at > 90% and was measured by trypan blue exclusion assay using ViCell-XR viability counter.

### Extracellular and intracellular antigen analysis of cell lines

A comprehensive surface antigen analysis using flow cytometry was performed. Surface antigens present on progenitor, immature, activated germinal center and memory B-cells along with those present on plasmablasts and plasma cells were examined. Presence of stem cell markers was also examined. Briefly, all cell lines were maintained in continuous culture at 37°C and 5% CO2 in a fully humidified incubator and were washed once with FCM buffer (PBS, 0.5% bovine serum albumin, 0.1% sodium azide and 0.004% Na4EDTA; Leinco Technologies Inc., Fenton, MO). Cells were suspended in FCM buffer at a concentration of 2 x 10^7^ cells/mL with purified human IgG (6 mg/ml, Sigma) to block binding of monoclonal antibodies (mAbs) to Fc receptors. After 10 minutes, 0.5–1 x 10^6^ cells were mixed with the indicated mAbs in a 12 x 75 mm tubes (Falcon, BD Bioscience, Bedford, MA). MAbs were purchased from BD Bioscience (San Jose, CA), Beckman Coulter (Miami, FL), Biolegend (San Diego, CA), Bio-Rad Serotec (Hercules, CA), Dako (Carpinteria, CA), eBiosciences (San Diego, CA), Life Technologies (Grand Island, NY) and used at a laboratory optimized saturating concentration. The cells were incubated in the dark with mAbs at ambient temperature for 20 minutes, then washed twice in FCM buffer and finally suspended in 2% methanol free formalin (Polysciences, Inc., Warrington, PA) and stored in the dark at 4–8°C no longer than 24 hours until analysis. For intracellular staining the cells were washed 15 minutes after the aforementioned fixation step in FCM buffer and then suspended in a 1:4 dilution of Reagent B (Fix& Perm cell fixation & permeabilization kit; Life Technologies) with the intracellular mAb of interest. The cells were incubated for 30 minutes before washing and suspending in FCM buffer for data acquisition. Cytofluorometric analysis was performed using an LSR Fortessa (BD BioSciences, San Jose, CA) flow cytometer calibrated daily with CS&T beads (BD Bioscience). This instrument was equipped with a 405, 488 and 640 nm solid state lasers. FITC, PE, PECy5 or PerCPCy5.5 were excited by the 488 nm laser and detected with 530/30 nm, 575/26 nm, 695/40 nm bandpass filters, respectively; APC was excited by the 640 nm laser and detected with a 670/34 nm bandpass filter. Thirty thousand events were collected using a forward scatter threshold. Data was analyzed using WinList (Verity Software House, Topsham, ME) using a broad forward versus side scatter region was used to include all cells while excluding any debris, dead cells and cell aggregates. Tumor cells were considered positive or negative for a given CD antigen/cell marker based on a cutoff of >20% gated expression or <20% gated expression, respectively. Spherotech 6 peak beads (Ultra Rainbow Calibration Particles; Lake Forest, IL) were used to convert mean fluorescence intensity (MFI) to molecules of equivalent soluble fluorochrome (MESF) according to the manufacturer’s instructions. Briefly, MFI and MESF values for each bead standard were log(10) transformed and used to calculate a best fit linear regression line at the same voltage settings used to acquire the cell line data. The regression line equation was then used to extrapolate the MESF values from the MFI of the mAb labeled cell line. Individual best-fit lines were determined for each fluorochrome. Qualitative classifiers based on MESF values were applied for surface marker density, denoting low (MESF >3,170), medium (MESF >33,014) and high (MESF >343,793) expression of CD antigens present on WM tumor cells. The full list of mAbs used is presented in S1 Data.

## Results

### Antigen expression profile of RPCI-WM1 cells

WM cells typically demonstrate detectable surface expression of IgM, monotypic surface light chain (most cases are κ^+^), CD19, and CD20 but do not express CD5, CD10, CD23.[1] However, a great degree of variability has been observed in the expression (or lack thereof) of these surface markers, as well as expression of atypical antigens.[6] In this regard, it is not uncommon for malignant B-cells to aberrantly express T-cell,[15–17] myeloid cell,[6, 18] and stem cell surface markers.[19–21] Using our recently established RPCI-WM1 model, we performed a comprehensive immunophenotype analysis to determine the expression of typical as well as unique CD antigens in these cells. Expression of 65 extracellular and 4 intracellular antigens, comprising B-cell, plasma cell, T-cell, NK-cell, myeloid and hematopoietic stem cell surface markers was analyzed by flow cytometry. Tumor cells were gated for antigen presence and a threshold of 20% was used to delineate relative expression (positive) vs. non-expression (negative) of antigen. RPCI-WM1 cells were positive for a total of 27 antigens and negative for 42 antigens (Table 1). Upon assessment of CD marker density where the antigen was expressed on >80% of cells, RPCI-WM1 cells were CD38+high (MESF 413,090), CD70+medium (MESF 49,943) and CD39+medium (MESF 30,127) with low to medium density (MESF 10,000–29,000) observed for CD28, CD43, CD45, CD54, CD138, CD184 and κ light-chain expression (Table 2). The finding of CD28 positivity was surprising yet not unfounded as a subset of long lived bone marrow plasma cells are known to use CD28 signaling for survival.[22] All remaining antigens expressed from Table 1 were either expressed at a low surface density or expressed on less than <20% of tumor cells.

**Table 1:** Expression of cell surface antigens on RPCI-WM1 model.

Expressed on >20% of cells		Expressed on <20% of cells		
CD14	CD134	CD5	CD80	CD117 (c-KIT)
CD22	CD272	CD10	CD90	CD127 (IL7Ra)
CD28	KAPPA	CD11c	CD111	CD135 (FLT3)
CD38	CD25 (IL2Ra)	CD13	CD133	CD137 (TNFRSF9)
CD39	CD54 (I-CAM1)	CD19	CD154	CD197 (CCR7)
CD43	CD123 (IL3R)	CD20	CD202b	CD243 (MDR1)
CD45	CD138 (Syndecan-1)	CD23	CD278	CD252 (TNFSF4)
CD45RA	CD184 (CXCR4)	CD24	CD16 (FcγRIIIa/b)	CD309 (VEFR2)
CD45RO	CD268 (BAFFR)	CD32	CD27 (TNFRSF7)	CD338 (ABCP)
CD66b	CD279 (PD-1)	CD34	CD30 (TNFRSF8)	CD62L (L-Selectin)
CD70	Intracellular-CD247	CD35	CD40 (TNFRSF5)	FMC7
CD86	Intracellular-CD289 (TLR9)	CD69	CD52 (CAMPATH-1)	HLA-DR
CD101	Intracellular-KAPPA	CD73	CD56 (NCAM-1)	LAMBDA
CD110		CD79b	CD105 (Endoglin)	

**Table 2:** Most widely expressed antigens on RPCI-WM1 cells.

mAb	Fluorophore	%Gated	MESF
Extracellular antigens			
CD28	APC	99.6	21596.2
CD38	PECY5	99.5	413090.1
CD39	PE	98.6	30127.2
CD43	FITC	97.9	27244.7
CD45	FITC	98.1	24447.2
CD45RO	PE	89.4	7268.5
CD54	FITC	98.3	22809.7
CD66b	PCPCY5.5	97.9	3592.2
CD70	PE	99.2	49943.4
CD86	APC	99.6	10598.5
CD110	PE	96.7	6712.5
CD123	PE	85.9	7301.2
CD134	PE	80.7	4237.7
CD138	PE	87.7	14582.8
CD184	PECY5	98.3	19115.2
KAPPA	APC	99.1	10789.6
**Intracellular antigens**			
LAMBDA	PE	87.8	6418.4
CD289	PE	99.4	21063.7
CD247	FITC	99.7	51184.7
KAPPA	FITC	99.8	360028.6

### Expression of WM-associated surface antigens present on RPCI-WM1

**Fig 1:**
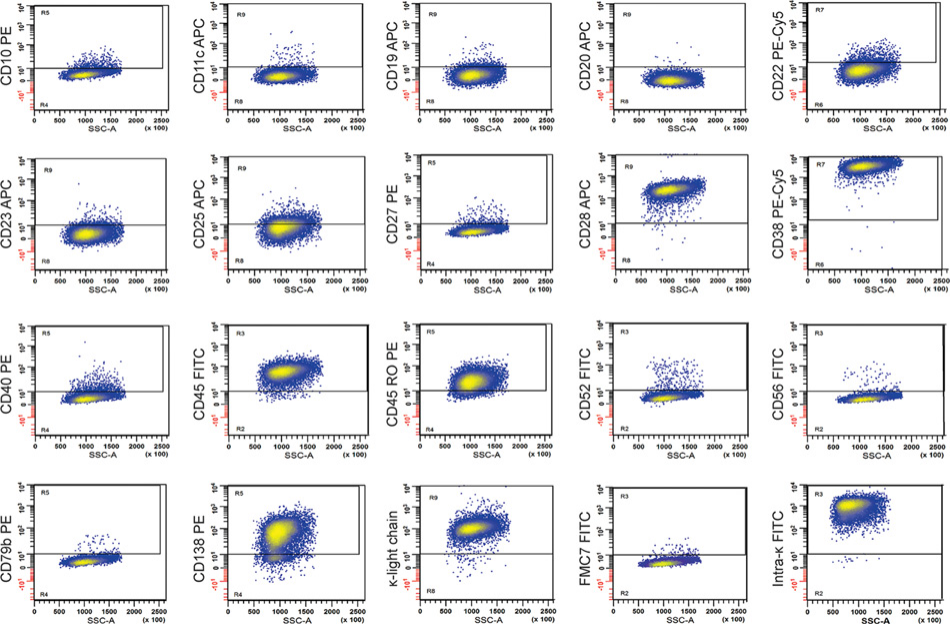
Expression of WM-specific antigens present on RPCI-WM1. For antigen detection, fluorescein (FITC), phycoerythrin (PE), phycoerythrin—cyanine 5 (PC5) or allophycocyanin (APC) conjugates of various antigen-specific antibodies were used. Flow cytometry shows the RPCI-WM1 cell line to be CD10, 11c, 19, 20, 23, 27, 40, 52 and 79b negative and CD22, 28, 38, 45, 45RO, 38, 138, κ light chain and intracellular-κ positive.

Next, we probed for antigens whose expression (or infrequent presence) on primary malignant cells from WM patients has been previously reported.[6, 8, 10] Nineteen extracellular and 1 intracellular surface marker(s) were examined showing RPCI-WM1 cells to be CD28+, CD38+, CD45+, CD45RO+, CD138+, κ light chain+ and intracellular κ light chain+ while being CD10-, CD11c-, CD19-, CD20-, CD22-, CD23-, CD25-, CD27-, CD40-, CD52-, CD56-, CD79b- and FMC7- (Fig 1). It is important to note that CD10, CD22 and CD23 have been reported as being expressed in 3%, 33% and 61% of patients.[6] The observation that these antigens were not expressed in the majority of RPCI-WM1 cells was not altogether unforeseen as this cell line was derived from a terminally advanced stage WM patient and suggests the shedding of “typical” surface antigens and upregulation of others (i.e. CD28) by the tumor clone, perhaps to retain its tumorigenicity.

### Comparison of the WM-associated antigens present on RPCI-WM1, BCWM.1 and MWCL-1

**Fig 2:**
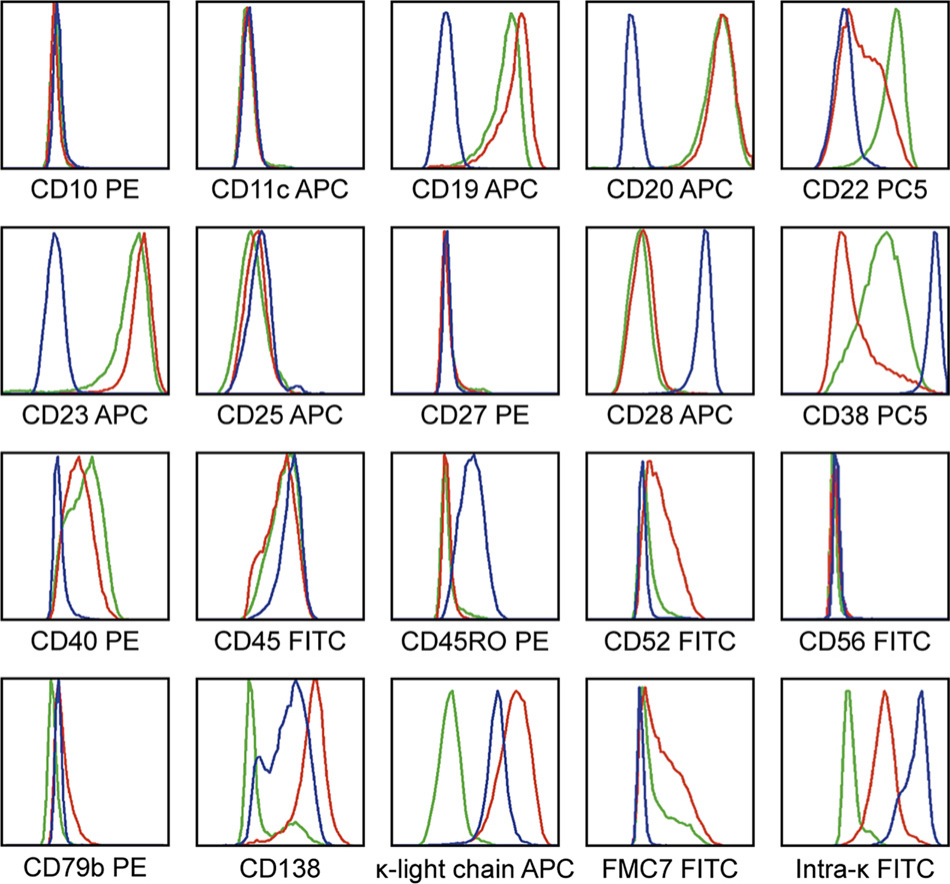
WM-specific antigen expression compared across RPCI-WM1, BCWM.1 and MWCL-1 cell lines. Fluorescein (FITC), phycoerythrin (PE), phycoerythrin—cyanine 5 (PC5) or allophycocyanin (APC) conjugates of various antibodies were used as presented above. All cell lines were negative for CD10 and 11c. Blue line indicates RPCI-WM1 antigen expression, red line indicates MWCL-1 antigen expression and green line indicates antigen expression in BCWM.1 cell line. Only BCWM.1 an MWCL-1 were CD19+, CD20+ and FMC7+. Expression of CD138 and κ-light-chain was seen only on MWCL-1 and RPCI-WM1 cells. CD28 expression was markedly more observable on RPCI-WM1 tumor cells as compared to MWCL-1 or BCWM.1.

We then conducted a comprehensive comparative analysis of surface markers in WM cell lines (only 3 noted in the medical literature, 2 developed by the Mayo Clinic group; MWCL-1 and RPCI-WM1,[10, 14] and 1 developed at Dana Farber Cancer Institute; BCWM.1).[13] Using the same set of 19 antigens (Fig 1) a comparative analysis was performed in BCWM.1 and MWCL-1 (Fig 2). Both MWCL-1 and BCWM.1 cells were CD19+low and CD20+medium. The CD20 epitope, FMC7, was also expressed in ~45–63% of BCWM.1 and MWCL-1 cell, respectively albeit at low levels. Notably both RPCI-WM1 (88.7% of cells) and MWCL-1 (98.3% of cells) were CD138+medium. RPCI-WM1 (99.1% of cells) demonstrated low density of κ light-chain (MESF 10,789.6) whereas density of κ light-chain on MWCL-1 (99.5% of cells) was medium in qualitative assessment (MESF 33,782). Contrastingly, in BCWM.1, CD138 was only expressed on 34.9% of gated cells at a low density (MESF 3,719) and κ light-chain on a minor fraction (26.7%) at a very low density (MESF 633.4). CD38 expression was most prominent in RPCI-WM1, followed by BCWM.1 (91.5% of cells, MESF 9,367.9) and least in MWCL-1 (44.4% of cells, MESF 2,108) (Table 3). In contrast to RPCI-WM1 cells, CD28 was expressed in <20% of BCWM.1 or MWCL-1 cells and whose surface density was very low. As anticipated, CD10 and 11c were not expressed in either of three cell lines.

**Table 3:** Pattern of surface antigen expression (>20%) across all 3 WM models.

		RPCI-WM1		MWCL-1		BCWM.1	
mAb	Fluorophore	% Gated	MESF	% Gated	MESF	% Gated	MESF
CD38	PECY5	99.5	413090.1	44.4	2108.0	91.5	9367.9
CD70	PE	99.2	49943.4	99.8	82511.4	97.2	64254.9
CD39	PE	98.6	30127.2	99.3	98989.5	99.2	61333.4
CD86	APC	99.6	10598.5	87.9	4830.0	98.3	12328.0
KAPPA	APC	99.1	10789.6	99.5	33782.1	26.7	633.4
CD54	FITC	98.3	22809.7	92.9	21078.1	97.2	43972.6
CD45	FITC	98.1	24447.2	84.8	12746.6	91.4	15611.4
CD43	FITC	97.9	27244.7	98.7	40373.8	94.4	31498.4
CD138	PE	87.7	14582.8	98.3	70921.5	34.9	3719.5
CD66b	PCPCY5.5	97.9	3592.2	93.8	3440.4	77.7	2273.3
CD110	PE	96.7	6712.5	97.9	9597.9	64.6	3687.2
CD123	PE	85.9	7301.2	89.8	12928.3	79.0	9597.9
CD134	PE	80.7	4237.7	67.3	4562.2	92.3	9828.2
CD268	PE	33.5	2526.3	94.9	32241.6	97.4	24368.2
CD45RA	APC	48.7	1087.7	50.6	1184.8	89.7	5577.1
CD101	APC	41.7	795.8	80.9	3632.9	35.3	535.8
CD25	APC	40.3	849.9	22.1	546.6	20.3	394.5
CD279	APC	39.0	774.2	23.5	274.7	24.6	579.2
CD272	PE	24.1	1981.1	96.1	31805.1	88.8	27111.1
CD22	PECY5	20.0	958.8	63.7	2819.4	97.3	15665.8
**INTRACELLULAR**							
KAPPA	FITC	99.8	360028.6	99.6	43972.6	67.2	6062.7
CD247	FITC	99.7	51184.7	99.2	20117.1	98.3	32369.7
CD289	PE	99.4	21063.7	99.3	10519.5	97.5	17236.6

### Pattern of surface antigen expression across all 3 WM models

**Fig 3:**
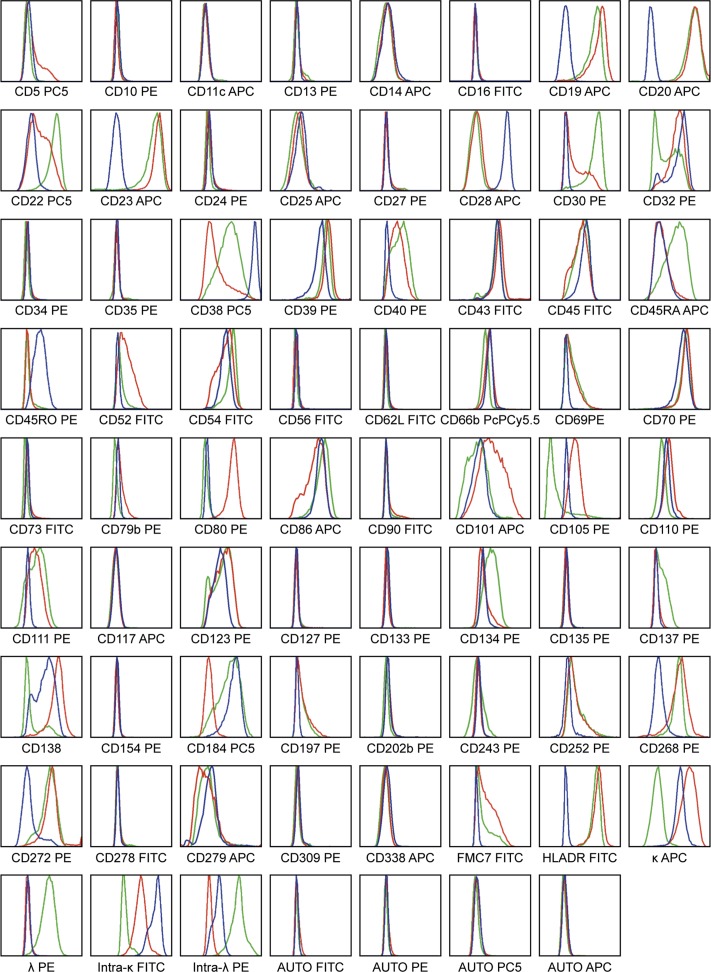
Comparative immunophenotyping analysis of RPCI-WM1, BCWM.1 and MWCL-1 WM cell lines. A total of 65 extracellular and 4 intracellular antigens, comprising B-cell, plasma cell, T-cell, NK-cell, myeloid and hematopoietic stem cell surface markers were analyzed by flow cytometry analysis. Quantification of % gated antigen expression and density of antigen expression is detailed in Table 4. Blue line indicates RPCI-WM1 antigen expression, red line indicates MWCL-1 antigen expression and green line indicates antigen expression in BCWM.1 cell line.

Overall antigen expression that was similarly present on at least 20% of malignant cells across all 3 WM models was analyzed. A total of 20 surface molecules were expressed on >20% of RPCI-WM1, BCWM.1 and MWCL-1 tumor cells (Table 3). Notably, CD39, 43, and 70 were found on more than 98% of cells across all 3 cell lines, exhibiting a medium density pattern of expression. CD45 was prominent on 84–98% of tumor cells from all cell lines with the highest density signal in RPCI-WM1 cells (MESF 24,447), nearly twice as high compared to BCWM.1 cells. In contrast, the long ~220kD isoform CD45RA was highest in BCWM.1 (89.7% of cells, MESF 5,577) and lowest on RPCI-WM1. CD134+low signal intensity was found on ~67%, 80% and 92% of MWCL-1, RPCI-WM1 and BCWM.1 cells, respectively. Markers that were found only on 20–40% of cells in a low density pattern included CD25, 66b and 279. Of the remaining antigens, CD86, although apparent on the surface of >85% of cells from all three tumor models, was most reactive (as based upon MESF) on RPCI-WM1 followed by BCWM.1 and least on MWCL-1 cells. The plasma cell surface marker CD138 was however most widely observed on MWCL-1 (98% of cells) and RPCI-WM1 (88% of cells), yet was found on only 35% of BCWM.1 cells. Expression of CD268/BAFFR (B-cell activating factor receptor) and CD272/BTLA (B and T lymphocyte attenuator) was also more apparent on MWCL-1 cells as well as BCWM.1, but negligible on RPCI-WM1. Lastly, expression of CD22, which functions as an inhibitory receptor for B-cell receptor signaling[23] was low on ~98% of BCWM.1 tumor cells (MESF 15,665.8), lesser so on 63% of MWCL-1 cells (MESF 2,819.4) and scarcely present on 20% of RPCI-WM1 cells (MESF 958.8). Additional data as well as a comprehensive immunophenotypic comparison across all 3 models is presented in Fig 3 and Table 4.

**Table 4:** Comprehensive immunophenotype comparison of RPCI-WM1, MWCL-1 and BCWM.1.

		RPCI-WM1		MWCL-1		BCWM.1	
WM associated antigens	Fluorochrome	% Gated	MESF	% Gated	MESF	% Gated	MESF
CD10	PE	8.8	1661.6	2.5	1057.6	4.5	1216.1
CD11c	APC	3.5	318.3	2.1	231.0	6.8	285.6
CD19	APC	9.0	394.5	97.8	30981.5	98.0	16617.6
CD20	APC	1.1	0.0	99.8	92499.2	98.9	66979.7
CD22	PECY5	20.0	958.8	63.7	2819.3	97.3	15665.7
CD23	APC	8.9	372.8	99.4	110591.5	94.8	38999.8
CD25	APC	40.3	849.9	22.1	546.6	20.3	394.5
CD27	PE	4.3	1374.9	8.5	1343.1	7.3	1661.5
CD28	APC	99.6	21596.2	16.0	427.1	12.0	329.2
CD38	PECY5	99.5	413090.1	44.4	2107.9	91.5	9367.8
CD40	PE	10.9	1661.6	79.8	5994.1	82.8	8448.2
CD52	FITC	4.8	2183.6	65.5	7335.7	20.9	3158.5
CD56	FITC	2.1	1814.0	2.2	1537.6	2.2	1353.8
CD138	PE	87.7	14582.8	98.3	70921.5	34.9	3719.5
CD45RO	PE	89.4	7268.5	4.5	1311.3	12.5	1470.3
CD79b	PE	1.6	1311.4	24.6	2109.1	2.0	491.1
FMC7	FITC	1.1	1583.7	63.6	8659.9	45.4	5827.4
KAPPA	APC	99.1	10789.6	99.5	33782.1	26.7	633.3
**All other antigens**	**Fluorochrome**	**% Gated**	**MESF**	**% Gated**	**MESF**	**% Gated**	**MESF**
CD5	PECY5	3.9	689.1	30.9	1317.3	4.0	288.5
CD13	PE	4.1	1534.1	6.1	1438.5	17.2	1629.7
CD14	APC	27.6	600.9	17.3	448.8	18.1	405.3
CD16	FITC	2.8	1767.9	2.0	1537.6	2.7	1583.6
CD24	PE	15.8	2109.2	15.2	1949.1	7.3	1343.1
CD30	PE	1.8	1279.6	50.5	4984.5	95.3	52990.2
CD32	PE	0.9	710.6	0.9	553.6	3.2	553.6
CD34	PE	4.8	1406.7	4.7	1216.1	5.4	994.3
CD35	PE	1.4	1216.1	2.2	1025.9	8.5	1184.3
CD39	PE	98.6	30127.2	99.3	98989.4	99.2	61333.3
CD43	FITC	97.9	27244.7	98.7	40373.8	94.4	31498.3
CD45	FITC	98.1	24447.2	84.8	12746.5	91.4	15611.4
CD45RA	APC	48.7	1087.7	50.6	1184.8	89.7	5577.0
CD54	FITC	98.3	22809.7	92.9	21078.1	97.2	43972.5
CD62L	FITC	2.5	1814.0	3.6	1675.7	1.5	1216.2
CD66b	PCPCY5.5	97.9	3592.2	93.8	3440.3	77.7	2273.3
CD69	PE	3.1	1311.4	46.0	3396.2	50.3	3525.5
CD70	PE	99.2	49943.4	99.8	82511.4	97.2	64254.8
CD73	FITC	2.7	1860.2	7.5	2044.9	0.2	6062.7
CD80	PE	2.4	1279.6	99.7	39474.6	3.8	804.9
CD86	APC	99.6	10598.5	87.9	4830.0	98.3	12327.9
CD90	FITC	2.6	1767.9	17.9	2739.9	10.1	1998.6
CD101	APC	41.7	795.8	80.9	3632.8	35.3	535.7
CD105	PE	6.4	1565.9	79.0	5114.6	12.5	0
CD110	PE	96.7	6712.5	97.9	9597.9	64.6	3687.1
CD111	PE	5.2	1470.4	66.0	4335.0	63.7	4594.6
CD117	APC	4.2	329.2	2.5	274.7	5.2	274.7
CD123	PE	85.9	7301.2	89.8	12928.3	79.0	9597.9
CD127	PE	2.3	1247.8	4.0	1152.6	7.2	1279.5
CD133	PE	4.8	1629.7	3.5	1247.8	7.4	1502.2
CD134	PE	80.7	4237.7	67.3	4562.1	92.3	9828.1
CD135	PE	6.5	1597.8	5.0	1343.1	8.2	1406.7
CD137	PE	4.7	1502.2	6.8	1406.7	52.4	3428.5
CD154	PE	1.3	1152.7	2.4	1025.9	4.2	1120.9
CD184	PECY5	98.3	19115.2	8.6	740.5	88.6	9618.4
CD197	PE	1.7	1216.1	41.5	3234.8	36.6	2719.2
CD202b	PE	12.1	1821.2	14.4	1885.1	9.0	1279.5
CD243	PE	16.3	2173.3	14.4	1949.1	26.3	1725.4
CD252	PE	16.6	1885.2	45.7	4952.0	38.4	5863.7
CD268	PE	33.5	2526.3	94.9	32241.5	97.4	24368.1
CD272	PE	24.1	1981.1	96.1	31805.0	88.8	27111.1
CD278	FITC	4.4	2137.4	5.9	2183.6	6.6	2461.5
CD279	APC	39.0	774.2	23.5	274.7	24.6	579.1
CD309	PE	11.8	1853.2	9.3	1629.7	8.7	1438.5
CD338	APC	12.1	470.6	5.4	329.2	6.8	285.6
HLADR	FITC	1.6	1721.8	99.6	149806.8	99.6	105318.4
LAMBDA	PE	4.1	1534.1	4.4	1311.3	98.1	20863.7

### Presence of stem-cell antigens on RPCI-WM1 cells and in comparison with BCWM.1 and MWCL-1

**Fig 4:**
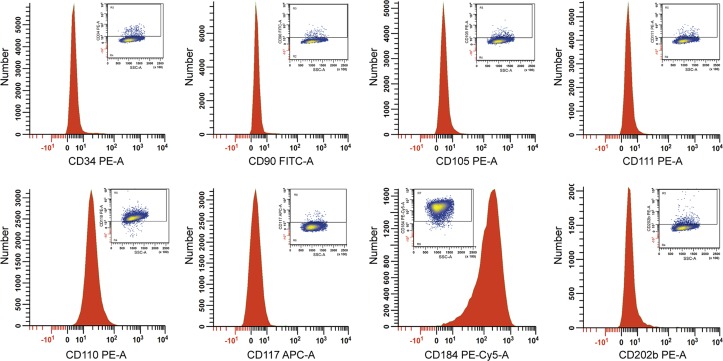
Presence of stem-cell markers on RPCI-WM1. A total of 8 surface antigens that are typically expressed on the surface of stem cells were examined. Notably, more than 90% of RPCI-WM1 cells gated were CD110+low and CXCR4/CD184+low. A comparison of these and the remaining stem-cell antigens in BCWM.1 and MWCL-1 cell lines is presented in Table 4.

Tumor cells from many diseases, including B-cell cancers, have been found to express surface markers that are more typically used to characteristic hematopoietic stem cells. As such, we first examined in RPCI-WM1, the expression of 8 stem cell markers whose expression has been shown on lymphoid or myeloid lineage cancers (Fig 4).[24–30] RPCI-WM1 cells were noted to be negative for CD34, 90, 105, 111, 117 and 202b while being positive (>95% of gated cells) for CD110 and CD184 (CXCR4). Both markers were lowly expressed with CD184 fluorescence being higher (MESF 19,1152) than CD110 (MESF 6,712). Expression of these antigens, as well as the 6 others that RPCI-WM1 cells did not express, were quantified in BCWM.1 and MWCL-1 cells also. While CD184 was expressed on >80% of BCWM.1 cells, its surface density was low (MESF 9,618). Contrastingly, CD184 was virtually absent on MWCL-1 cells (8.8% cells gated, MESF 740.5). We did not observe any expression of CD34, 90 or 202b on either cell line, however, CD105+low expression was found on approximately 79% of MWCL-1 cells but virtually absent, on BCWM.1, similar to RPCI-WM1. Also, CD111 was lowly expressed on >60% of BCWM.1 (MESF 4,594.6) and MWCL-1 tumor cells (MESF 4,335) in contrast to 5.2% of RPCI-WM1 (MESF 1,470.4) (see Table 4).

### Immunophenotype comparison of WM models with other B-cell cancers

WM cells exist within a continuum comprised of morphological (and functional) features belonging to both B-cells and plasma cells, carrying a marker expression pattern that can sometimes mimic those of other B/plasma cell malignancies. We compared the expression of 11 surface markers that either alone or in combination with one another are typically observed in patients with WM, chronic lymphocytic leukemia (CLL), mantle cell lymphoma (MCL), splenic marginal zone lymphoma (SMZL), follicular lymphoma (FL), hairy cell leukemia (HCL), mucosa associated lymphoid tissue (MALT) lymphoma and multiple myeloma (MM) with those expressed on the 3 WM cell lines (Table 5).[6, 31, 32] We noted that CD11C, which has been shown in up to 81% of WM cases examined and is also expressed in SMZL, MCL, FL, HCL and MALT lymphoma was absent in the 3 available WM models.[6] We also found that approximately 30% of MWCL-1 cells expressed CD5+low, which has been shown in a very small subset of WM cases (~5%),[6] however, this marker was not found on BCWM.1 and RPCI-WM1. Despite overlap in antigen expression, Table 5 shows distinctions between surface molecules present on common B/plasma cell cancer cases (including WM) compared with the 3 WM cell lines.

**Table 5:** Immunophenotype comparison of common WM models with various B/plasma cell cancers.

	B/plasma cell malignancies								WM models		
Antigen	LPL/WM	SMZL	CLL	MCL	FL	HCL	MALT	MM	RPCI-WM1	BCWM.1	MWCL-1
CD5	-	+	+	+	-	-	-	-	-	-	+
CD10	-	-	-	-	+	-	-	-	-	-	-
CD11C	+	+	-	+	+	+	+	-	-	-	-
CD19	+/-	+	+	+/-	+/-	-	+	-	-	+	+
CD20	+/-	+	+	+	+	+	+	-	-	+	+
CD23	+/-	+	+	-	+	-	-	+/-	+	-	+
CD25	+/-	+	-	-	-	+	-	-	+	+	+
CD27	+/-	+	+	+	+	-	+	-	-	-	-
CD38	+	+/-	+	+	+	-	+/-	+	+	+	+
CD138	+	+/-	-	-	-	-	+/-	+	+	+	+

Abbreviations: +, present in >20% gated cells;-, present in <20% of gated cells; LPL/WM, lymphoplasmacytic lymphoma/Waldenström macroglobulinemia; SMZL, splenic marginal zone lymphoma; CLL, chronic lymphocytic leukemia; MCL, mantle cell lymphoma; FL, follicular lymphoma; HCL, hairy cell leukemia; MALT, mucosa-associated lymphoid tissue; MM, multiple myeloma.

## Discussion

The fitness of a preclinical model to faithfully reproduce a testable phenotype of interest (i.e. drug sensitivity) relies on an intimate knowledge of the underlying molecular architecture as well as its immunophenotypic features. Establishment of online databases such as the Cell Line Encyclopedia (Broad Institute, MA) demonstrates the extensive characterization efforts currently underway to define the genomic features for a large number of tumor cell lines.[7] However, no such initiatives have as yet been made in describing the molecular attributes of WM cell line models, particularly as they pertain to the immunophenotype profile. As such, we conducted a comprehensive surface antigen analysis as well genomic and epigenetic inspection (forthcoming report) of the three most used WM cell lines: BCWM.1, MWCL-1 and RPCI-WM1.

WM is a unique pathologic entity with well-defined clinical features. However, a significant degree of heterogeneity is observed in patients with this disease, where some are asymptomatic and require no therapy, while others necessitate therapeutic intervention for management of symptomatic disease.[2] This heterogeneity is further reflected within the WM tumor compartment, which consists of B-cells, plasma cells and lymphoplasmacytic cells and whose spatiotemporal profile can shift under prolonged therapeutic stress. A growing body of evidence suggests that as WM patients are treated with chemotherapy (fludarabine, cyclophosphamide, vincristine) or immunotherapeutics (anti-CD20 monoclonal antibody, rituximab), the B-lymphocytic WM component is effectively eradicated, leaving behind the more robust plasma cell (or plasmacytoid) fraction, which is known to be considerably less sensitive to chemotherapy or anti-CD20 therapy.[9, 33–35] In one series, persistent plasmacytosis was observed in 24% of patients following therapy, occurring as early as 1 month post-treatment in one patient and in a separate case, enduring as late as 50 months post-treatment.[8] In this regard, appropriate in vitro and in vivo preclinical models are needed to study the variance in immunophenotypic changes as well as their potential clinical ramifications.

Although more descriptive in nature, our current analysis suggests that the most commonly used WM cell lines models each possess unique immunophenotypic features; reminiscent conceivably of the tumor cell populations from which they were derived. The RPCI-WM1 cell line in particular represents a unique model amongst the three, due to expressional loss of CD19, CD20, CD23 and enhanced expression of CD28 and CD184. Importantly, the index patient from whom RPCI-WM1 was developed had previously received two lines of rituximab containing therapy, which she subsequently developed resistance towards. It is therefore not surprising that the malignant cells fit enough to establish themselves as the dominant WM clone lack CD20. Also, the increased expression and density of CXCR4 in RPCI-WM1 (and to a lesser extent BCWM.1; MESF/density, 9,618.5) is noteworthy as both mAb-based and small molecule inhibitors of CXCR4/CD184 are currently being examined for therapeutic use in a wide range of maladies.[36–38] The fully humanized anti-CXCR4 antibody, BMS-936564/MDX-1338 has shown preclinical efficacy in WM cell lines and is being tested in a phase I clinical trial for the treatment of relapsed/refractory hematologic cancers.[37, 39] In addition to CD184, we observed increased expression and density of CD38 on RPCI-WM1 cells compared to the other cell lines. This carries potential therapeutic implications as the fully human mAb, daratumumab, which targets CD38 has demonstrated impressive activity in relapsed/refractory multiple myeloma patients and holds promise in other CD38-expressing malignancies, including WM.[40, 41] Recognizing that in vitro models of disease may alter their molecular (and immunophenotypic) makeup during culture to sustain stable growth, we examined for the presence of novel (CD28), therapeutically relevant (CD38 and CD184/CXCR4) and established WM cell markers (CD19 and CD20) in CD19+/CD138+ sorted tumor cells derived ex vivo from WM patients (S1 Fig). As anticipated, we observed high expression of CD38 (>90%) and CD184 (>70%) in tumor cells from both patients however, CD28 expression was observed in <10% of gated tumor cells from either patient (S1 and S2 Tables). Contrasting against the high expression of CD28 in RPCI-WM1 cells, this finding was not wholly unexpected as RPCI-WM1 cells were established from a highly drug refractory and terminal WM patient, whereas, the primary tumor cells tested herein are from patients with a less aggressive disease course (see S1 Data). It is also conceivable that the in vitro stability of RPCI-WM1 cells was ultimately established from a CD28+ tumor clone, which was present as only a minority fraction in the index patient. Nevertheless, longitudinal studies examining CD28 expression in WM patients from the time of diagnosis till terminal stage disease are required to fully determine the correlation between CD28 expression and clinical progression of WM.

Although BCWM.1, MWCL-1 and RPCI-WM1 are the most routinely used human WM cell lines, a clonal relationship to the tumor cells of the index patient has only been demonstrated by molecular means (sequence analysis of IGHV/CDR3 length analysis) in the latter two models.[10, 11, 14] A direct clonal connection between BCWM.1 and the patient from which it was reportedly derived, has been a source of debate,[42, 43] yet we posit that sufficient evidence exists to support its use as an in vitro surrogate of human WM for the following reasons: 1.) BCWM.1 contains a heterozygous mutation in the myeloid differentiation primary response protein 88 gene (MYD88_L265P_), which is found in 90–97% of WM patients,[44, 45] and 2.) BCWM.1 expresses a wide variety of surface antigens (CD19, 20, 22, 23, 25, 38, 52, 138 and FMC7) that are typically present on WM cells from patients with the disease.[6] Thus, it is of great importance to fully characterize the molecular and immunophenotypic features of these 3 distinct WM cell lines; in the process helping to define their optimal role in testing of targeted anti-WM therapeutics and also shedding biological insight into their differential drivers.

Future studies examining distinct cellular sub-populations within RPCI-WM1 itself (as well as the BCWM.1 and MWCL-1 models) by multiparameter gating strategies will explicate the precise significance of these differentially expressed markers and their function in WM biology.

## Supporting Information

S1 Data Supplementary Materials & Methods. The dataset contains the Supplementary Materials & Methods.
(DOCX)

S1 Fig Expression of selected surface antigens present on CD19+/CD138+ sorted, primary patient derived WM tumor cells. For antigen detection, fluorescein (FITC) or phycoerythrin (PE) conjugates of various antigen-specific antibodies were used. Flow cytometry shows both WM patient 1 (WM1) and WM patient 2 (WM2) to be CD19, 20, 38 and 184 positive and negative for CD28. Percentage of cells positive and their Mean Fluorescent Intensity (MFI) are presented in S1 and S2 Tables, respectively.
(TIFF)

S1 Table % Gated expression of selected surface markers in primary WM tumor cells from patients (WM1 and WM2). Patient-derived tumor cells were studied by flow cytometry for expression of CD19, 20, 28, 38 and 184. Table shows the percentage of cells that were positive for the above tumor makers.
(DOCX)

S2 Table Mean Fluorescent Intensity (MFI) of selected surface markers in primary WM tumor cells from patients (WM1 and WM2). Patient-derived tumor cells were studied by flow cytometry for expression of CD19, 20, 28, 38 and 184. Table shows the MFI values for the above tumor markers.
(DOCX)
