# Supplementary material for: Immunophenotyping of Waldenströms Macroglobulinemia Cell Lines Reveals Distinct Patterns of Surface Antigen Expression: Potential Biological and Therapeutic Implications
Source: PLoS One. 2015 Apr 8;10(4):e0122338. doi: 10.1371/journal.pone.0122338 (PMC4390194; doi:10.1371/journal.pone.0122338)
Supplement: S1 Data — The dataset contains the Supplementary Materials & Methods. (DOCX) [file pone.0122338.s001.docx]

**Supplementary Materials & Methods.**

**Primary patient derived WM tumor cells**

CD19+/CD38+ sorted tumor cells obtained from consenting WM patients (n=2; WM patient 1 being relapsed and refractory and WM patient 2 currently receiving first line of treatment) were acquired from the Predolin Biobank (Mayo Clinic, Rochester MN) following approval by the Mayo Clinic Institutional Review Board.

**Extracellular antigen analysis of primary patient derived WM tumor cells**

For staining of cell surface markers, tumor cells from WM patients were washed twice with cold PBS and suspended in 300uL of binding buffer (PBS solution with 2% FBS). Cell were divided in three tubes; unstained, isotype control, and those with antibody against CD19, CD20, CD28, CD38 (Invitrogen, Life Technologies, Carlsbad CA) and CD184/CXCR4 (BioLegend, San Diego CA). 5uL of the respective antibody was added and cells were incubated for 30min at room temperature. Tumor cells were washed twice with cold PBS and suspended in 100uL of 4% Paraformaldehyde in PBS solution (Affymetrics Inc., 19943 1LT) followed by analysis using a BD Accuri TM C6 Flow cytometer. FCS Express 4 (Denovo softwares) was used to analyze the data.

**Full list of monoclonal antibodies used (mAbs) used in analysis of surface markers present on cell lines**

| **mAb** | **Vendor** | **Clone** | **Fluor** |
| --- | --- | --- | --- |
| CD10 | eBiosciences | SN5c | PE |
| CD101 | Biolegend | BB27 | APC |
| CD105 | BD Bioscience | 266 | PE |
| CD111 | Beckman Coulter | R1.302.12 | PE |
| CD117 | Beckman Coulter | IM3638 | APC |
| CD11c | BD Bioscience | S-HCL-3 | APC |
| CD123 | BD Bioscience | 9F5 | PE |
| CD127 | Beckman Coulter | R34.34 | PE |
| CD13 | BD Bioscience | L138 | PE |
| CD110 | BD Bioscience | 1.6.1 | PE |
| CD134 | Biolegend | Ber-ACT35 | PE |
| CD135 | BD Bioscience | 468 | PE |
| CD137 | BD Bioscience | 4B4-1 | PE |
| CD138 | Beckman Coulter | B-A38 | PE |
| CD14 | Life Technologies | TUK4 | APC |
| CD154 | BD Bioscience | 89-76 | PE |
| CD16 | Invitrogen | 3G8 | FITC |
| CD184 | BD Bioscience | 12G5 | PECY5 |
| CD19 | BD Bioscience | SJ25C1 | APC |
| CD197 | BD Bioscience | 3D12 | PE |
| CD20 | BD Bioscience | L27 | APC |
| CD202b | R&D Systems | Ala22-Gln760 | PE |
| CD22 | Beckman Coulter | SJ10.1H11 | PECY5 |
| CD23 | eBiosciences | EBVCS2 | APC |
| CD24 | BD Bioscience | ML5 | PE |
| CD243 | BD Bioscience | 15D3 | PE |
| CD25 | BD Bioscience | 2A3 | APC |
| CD252 | BD Bioscience | Ik-1 | PE |
| CD27 | BD Bioscience | L128 | PE |
| CD272 | BD Bioscience | J168-540 | PE |
| CD278 | eBiosciences | ISA-3 | FITC |
| CD279 | BD Bioscience | MIH4 | APC |
| CD28 | BD Bioscience | CD28.2 | APC |
| CD30 | BD Bioscience | Ber-H83 | PE |
| CD309 | BD Bioscience | 89106 | PE |
| CD32 | Life Technologies | AT10 | PE |
| CD338 | BD Bioscience | 5D3 | APC |
| CD34 | Beckman Coulter | 581 | PE |
| CD35 | BD Bioscience | E11 | PE |
| CD38 | Beckman Coulter | LS198-4-3 | PECY5 |
| CD40 | BD Bioscience | 5C3 | PE |
| CD45 | BD Bioscience | 2D1 | FITC |
| CD45RA | eBiosciences | HI100 | APC |
| CD45RO | Beckman Coulter | UCHL.1 | PE |
| CD5 | Beckman Coulter | BL1a | PECY5 |
| CD52 | Serotec | YTH34.5 | FITC |
| CD54 | Life Technologies | MEM-111 | FITC |
| CD56 | BD Bioscience | NCAM16.2 | FITC |
| CD62L | Dako | Y2/51 | FITC |
| CD66b | Biolegend | G10F5 | PCPCY5.5 |
| CD69 | BD Bioscience | L78 | PE |
| CD70 | BD Bioscience | Ki24 | PE |
| CD73 | eBiosciences | AD2 | FITC |
| CD79b | Life Technologies | SN8 | PE |
| CD80 | Beckman Coulter | MAB104 | PE |
| CD86 | BD Bioscience | 2331(FUN-1) | APC |
| CD90 | Beckman Coulter | F15-42-1-5 | FITC |
| FMC7 | BD Bioscience | FMC7 | FITC |
| HLADR | BD Bioscience | L243 | FITC |
| KAPPA | BD Bioscience | TB28-2 | APC |
| LAMBDA | BD Bioscience | 1-155-2 | PE |
| CD133 | Miltenyi Biotec | AC133 | PE |
| CD39 | BD Bioscience | TU66 | PE |
| CD43 | BD Bioscience | 1G10 | FITC |
| CD268 | BD Bioscience | 11C1 | PE |
|  |  |  |  |
| **INTRACELLULAR MARKERS** | |  |  |
| CD247 | Biolegend | 6B10.2 | FITC |
| CD289 | BD Bioscience | eB72-1665 | PE |
| KAPPA | BD Bioscience | TB28-2 | FITC |
| LAMBDA | BD Bioscience | 1-155-2 | PE |
