# Supplementary figures and images for: Immunophenotyping of Waldenströms Macroglobulinemia Cell Lines Reveals Distinct Patterns of Surface Antigen Expression: Potential Biological and Therapeutic Implications
Source: PLoS One. 2015 Apr 8;10(4):e0122338. doi: 10.1371/journal.pone.0122338 (PMC4390194; doi:10.1371/journal.pone.0122338)

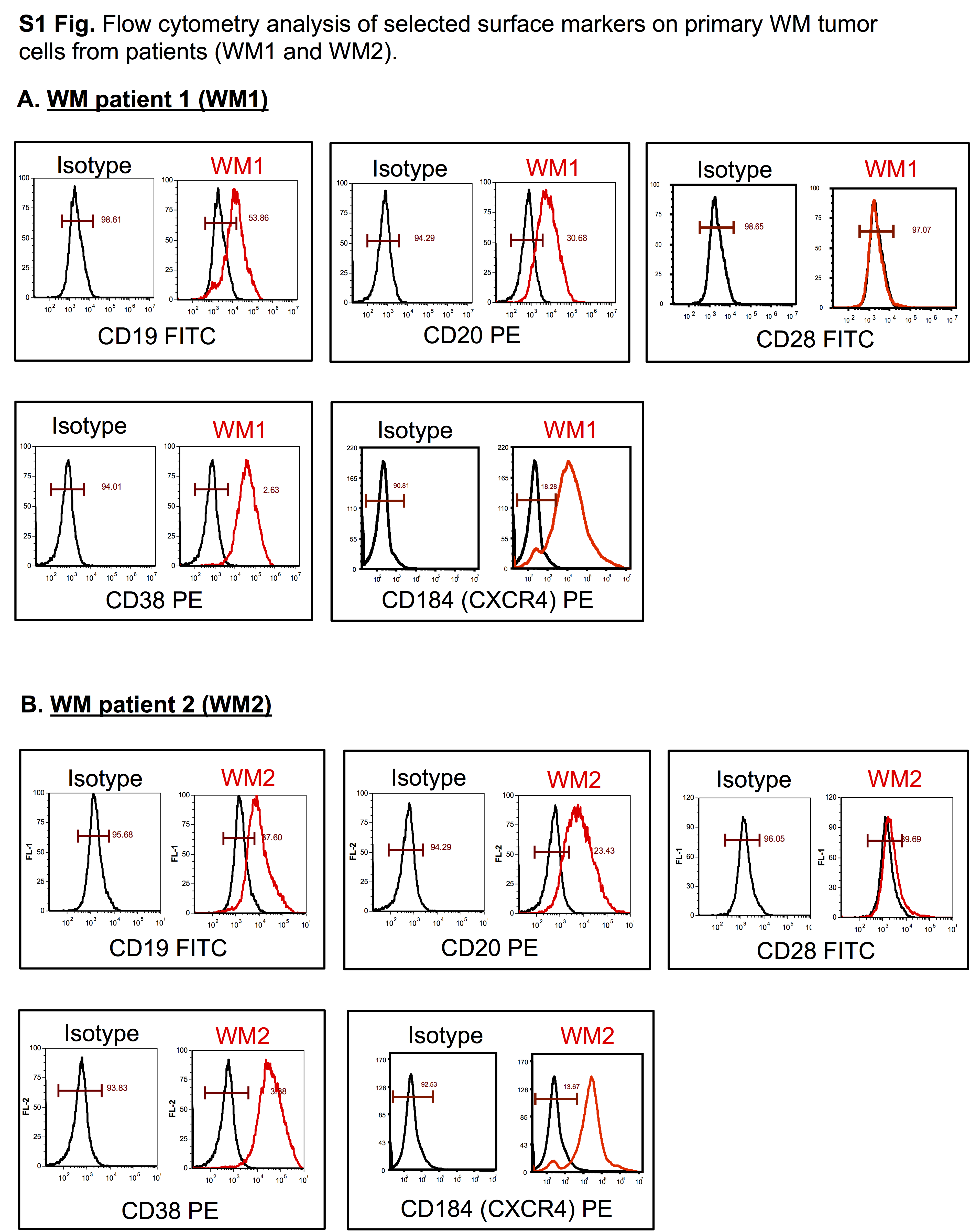

Supplement: S1 Fig — For antigen detection, fluorescein (FITC) or phycoerythrin (PE) conjugates of various antigen-specific antibodies were used. Flow cytometry shows both WM patient 1 (WM1) and WM patient 2 (WM2) to be CD19, 20, 38 and 184 positive and negative for CD28. Percentage of cells positive and their Mean Fluorescent Intensity (MFI) are presented in S1 and S2 Tables, respectively. (TIFF) [file pone.0122338.s002.tiff]
