# Supplementary material for: Immunophenotyping of Waldenströms Macroglobulinemia Cell Lines Reveals Distinct Patterns of Surface Antigen Expression: Potential Biological and Therapeutic Implications
Source: PLoS One. 2015 Apr 8;10(4):e0122338. doi: 10.1371/journal.pone.0122338 (PMC4390194; doi:10.1371/journal.pone.0122338)
Supplement: S1 Table — Patient-derived tumor cells were studied by flow cytometry for expression of CD19, 20, 28, 38 and 184. Table shows the percentage of cells that were positive for the above tumor makers. (DOCX) [file pone.0122338.s003.docx]

**S1 Table.** % Gated expression of selected surface markers in primary WM tumor cells from patients (WM1 and WM2).

| **% Gated expression** | | | | | |
| --- | --- | --- | --- | --- | --- |
|  | **CD19** | **CD20** | **CD28** | **CD38** | **CD184/CXCR4** |
| **WM1** | 44.75 | 63.61 | 1.58 | 91.38 | 72.53 |
| **WM2** | 58.08 | 70.86 | 6.36 | 90.45 | 78.86 |
