# Supplementary material for: Immunophenotyping of Waldenströms Macroglobulinemia Cell Lines Reveals Distinct Patterns of Surface Antigen Expression: Potential Biological and Therapeutic Implications
Source: PLoS One. 2015 Apr 8;10(4):e0122338. doi: 10.1371/journal.pone.0122338 (PMC4390194; doi:10.1371/journal.pone.0122338)
Supplement: S2 Table — Patient-derived tumor cells were studied by flow cytometry for expression of CD19, 20, 28, 38 and 184. Table shows the MFI values for the above tumor markers. (DOCX) [file pone.0122338.s004.docx]

**S2 Table.** Mean Fluorescent Intensity (MFI) of selected surface markers in primary WM tumor cells from patients (WM1 and WM2).

| **Mean Fluorescent Intensity (MFI)** | | | | | |
| --- | --- | --- | --- | --- | --- |
|  | **CD19** | **CD20** | **CD28** | **CD38** | **CD184/CXCR4** |
| **WM1** | 13136 | 6322 | 1966 | 40803 | 11958 |
| **WM2** | 8169.5 | 6084 | 2095 | 36595 | 24229.5 |
